# Contaminants in Human Milk: Weighing the Risks against the Benefits of Breastfeeding

**Published:** 2008-10

**Authors:** M. Nathaniel Mead

When it comes to feeding the newborn, human milk is, from an evolutionary perspective, the biological norm, the time-tested standard of care. The health benefits to the infant of breast-feeding have been amply documented; numerous studies strongly indicate significantly decreased risks of infection, allergy, asthma, arthritis, diabetes, obesity, cardiovascular disease, and various cancers in both childhood and adulthood. Among the more fundamental disadvantages of not being breastfed is a loss of immunologic protection afforded by maternal colostrum, a “pre-milk” fluid secreted only during the first days after delivery, as well as numerous other bioactive factors that help protect the infant through the first two years of life, when the immune and nervous systems are incompletely developed. Nevertheless, given the tendency for persistent organic pollutants (POPs), pesticides, heavy metals, and other contaminants to accumulate in human milk, researchers and parents alike are asking whether the nursling’s exposure to these pollutants might reduce or even override the health benefits.

## Veering Off the Evolutionary Path

Throughout primate evolution and pre-industrial human history, breastfeeding was the rule: the mother carried her baby and breastfed on demand. According to nutritional anthropologist Daniel W. Sellen in the 2007 edition of the *Annual Review of Nutrition*, breastfeeding beyond age 2 years was typical in 75–83% of hunter–gatherer societies, with the average age at weaning approximately 30 months. Moreover, copious data now support the hypothesis that humans evolved to begin consuming foods besides mother’s milk at approximately 6 months of age (Sellen also notes humans are the only primates that wean their infants before they can forage for themselves). This pattern was probably the norm for 200,000 years of human evolution and some 7 million years of nonhuman primate evolution.

A radical change occurred in the late 1800s, with the widespread relocation of rural populations to urban areas resulting in lifestyle and socio-cultural changes that disrupted the normal breastfeeding pattern. In a historical overview published in the December 2003 issue of the *American Journal of Public Health*, Ohio University sociologist Jacqueline H. Wolf described how large numbers of women in all echelons of European and U.S. society, prompted by different socioeconomic and cultural factors, began to supplement their own milk with cows’ milk soon after giving birth. Some avoided breastfeeding altogether, and those who did breastfeed increasingly weaned their babies before 3 months of age.

Then, in the early 1900s, U.S. public health officials began to report that “hand feeding” infants with unhygienically processed cow’s milk was spawning an epidemic of infant death and disease. In Chicago, for example, nearly 1 in 5 babies died before their first birthday, mainly from diarrhea, and for every breastfed baby that died there were 15 deaths from hand feeding. As part of a public health campaign to lower infant mortality, posters were mounted throughout U.S. cities urging mothers to breastfeed.

By the late 1920s, laws in most municipalities mandated that cow’s milk be processed under sanitary conditions, and pasteurized milk was hailed as safe for young and old alike. Despite continued warnings by public health officials on the hazards of artificial feeding, efforts to educate new and expecting mothers waned.

At the same time, more women began having their babies in hospitals rather than at home. Mothers and infants increasingly were separated as a matter of course after delivery, due to the rising use of anesthesia during labor, among other factors. Prolonged separation after birth can make it more difficult to establish breastfeeding; a Japanese study published by Nakao et al. in the January 2008 *International Breastfeeding Journal* showed that women who breast-fed their infants within 2 hours of birth were more than twice as likely to still be breastfeeding at 4 months compared with mothers who initiated breastfeeding more than 2 hours after birth.

Over the next few decades, the increasing availability of “milk substitutes” meant that more working-class women could enter the workplace sooner or devote more time to personal pursuits. “By 1971, breastfeeding had reached an all-time low in the United States. Only 24% of mothers initiated breastfeeding—that is, only 24% breastfed at least once before hospital discharge,” wrote Wolf in the *American Journal of Public Health*. Since then, she reported, breastfeeding rates have “inexplicably receded and surged.”

Today, the prevalence of initial breastfeeding among U.S. mothers is about 71%, according to a report in the 3 August 2007 *Morbidity and Mortality Weekly Report*, but only 11–14% of infants are exclusively breastfed (i.e., consume nothing else, including water) in the first 6 months, as recommended by the American Academy of Pediatrics and the World Health Organization (WHO). Only 16% of U.S. infants are still breastfeeding at 1 year of age; probably far fewer go on to breastfeed for the 2 years recommended by the WHO.

Figures in the February 2005 issue of *Public Health Nutrition* point to wide variation across the few European countries for which breastfeeding data are available. Initiation rates range from 63 to 99%, exclusive breastfeeding at 6 months ranges from 1 to 46%, and breast-feeding at 12 months ranges from 4 to 36%, with Nordic countries consistently showing the highest rates at each point.

Meanwhile, in many developing countries, the length of time babies are completely breastfed remains low. For example, in African countries about one-quarter of mothers exclusively breastfeed for 6 months, according to WHO figures. Yet a study reported by Edmond et al. in the March 2006 issue of *Pediatrics* found that 16% of all neonatal deaths in Ghana could be prevented if infants were breastfed from day one, 22% if breastfeeding started within the first hour after birth.

Maternal employment can be a major limiting factor in terms of breastfeeding duration. A study by Joan Y. Meek in the April 2001 *Pediatric Clinics of North America* found that only 10% of full-time working mothers provided any breast milk to their 6-month-olds, compared with almost 3 times that number of stay-at-home mothers; this pattern was consistent across all ethnic, educational, and age groups.

In the years since that study was published, numerous employers have established lactation rooms and breastfeeding-supportive workplace policies, and such efforts appear to be paying off. In the September–October 2006 issue of *Women’s Health Issues*, Ryan et al. reported that 26.1% of mothers studied who worked full-time and 36.6% of mothers who worked part-time were still breastfeeding at 6 months. The authors also reported that breastfeeding trends since 1984 indicated a more than 200% increase in the rate of breastfeeding at 6 months after delivery among full-time working mothers. However, these numbers still fall short of the federal Healthy People 2010 goal of 50% of mothers breastfeeding at 6 months.

## Human Milk: Its Own Immune System

One of the features unique to primate infants is slow early development of the immune system, during which time energy and nutrients are devoted to the growth and development of other systems such as the central nervous and musculoskeletal systems. According to Sellen, lactation is thought to have evolved around 200 million years ago as a means of transferring the protective functions of fully mature immune systems across generations; all mammals derive essential protection from their mothers’ milk.

“The mother supports the host defense of the infant in two ways,” says Lars Hanson, a clinical immunologist at Göteborg University in Sweden. “One is via antibodies from her blood that are actively transported over the placenta to the infant’s circulation during fetal life, and are ready for use from birth on. The other is due to the numerous and complex defense factors provided via the mother’s milk, available directly after delivery.”

The factors provided through mother’s milk not only effectively defend against many pathogens, but do so in a noninflammatory way, says Armond Goldman, an emeritus professor of pediatrics at The University of Texas Medical Branch in Galveston. By preventing inflammation, he adds, the integrity of the digestive and respiratory systems is preserved to ensure normal nutrition, growth, and functioning overall.

The noninflammatory and probiotic properties of human milk also help ensure that the infant’s intestinal tract will not be permeable to enteric pathogens. “This latter effect on the infant’s intestinal tract enables the infant to become actively immune to environmental pathogens, but without displaying overt signs of infection or inflammation,” says Goldman.

The composition of human milk undergoes remarkable quantitative changes as lactation proceeds, many of which track with changes in the developmental status of the infant. Human milk contains a rich array of proteins, carbohydrates, lipids, fatty acids, minerals, and vitamins, but most of its disease-fighting potential comes from a plethora of antibodies, leukocytes, hormones, antimicrobial peptides, cytokines, chemokines, and other bioactive factors that may be crucial to the infant’s defense against common pathogens in the first few weeks and months of life. Indeed, says Goldman, the effects of the immune system in human milk last for as long as the infant is breast-feeding and possibly beyond weaning.

**Table:** Immunoprotective Components of Breast Milk

Component	Property
Secretory IgA (sIgA)	Maternal-specific immunoglobulins to environmental antigens
Lactoferrin	Bacteriostatic, iron-binding protein; antiviral properties
Lysozyme	Bacteriocidal and anti-inflammatory activity; acts synergistically with peroxide and ascorbate to destroy *Escherichia coli* and some *Salmonella* strains
Bifidus factor	Promotes growth of beneficial *Lactobacillus bifidus* and low pH of stools; inhibits growth of *Shigella*, *Salmonella*, and some *E. coli* strains
Oligosaccharides	Complex carbohydrate moiety; block antigen attachment to gut epithelial receptors
Milk lipids	Antiviral, antibacterial, antiprotozoal properties
Milk leukocytes	Phagocytosis of bacteria, viruses, and fungi; secretion of numerous bioactive substance

**Adapted from:** Wagner CL, Anderson DM, Pittard WB. 1996. Special properties of human milk. *Clin Pediatr (Phila)* 35(6):283–293.

Among the more intriguing immune connections that have come to light is the so-called enteromammaric link. At birth, the newborn emerges from the sterile and protected environment of the mother’s uterus into a world teeming with microbes. The newborn’s gut and skin are “colonized” by whatever microbes he or she first comes into contact with. Ideally, this first exposure is to the mother’s own gut flora during vaginal birth; the child has already received antibodies to her microbes *in utero*, and the antibodies later provided via the mother’s milk continue to provide the precise protection the infant needs to fend off potential pathogens in the mother's gut flora. “Being delivered next to the mother’s anus, the newborn is subsequently colonized by the mother's microbial flora, but these flora are the least threatening, because mother’s milk affords protection against them,” explains Goldman. “In addition, some protection comes from her transplacentally transferred IgG [immunoglobulin G] antibodies, which have a more proinflammatory activity.”

A surge of knowledge about the immune system that began in the 1950s would eventually culminate in a radical reframing of the biological role of human milk. In volume 15, issue 4–5 (1959) of the *International Archives of Allergy and Applied Immunology*, Hanson coauthored a report describing antibodies in human milk that were active against many enteric bacteria and viruses. Two years later, Hanson isolated secretory immunoglobulin A (SIgA), the dominant immunoglobulin in the human body. SIgA turns out to be critical to maintaining mucosal immunity along the digestive and respiratory tracts, thus helping to explain breastfeeding’s protective effects against infections and allergies.

Recent research indicates that this milk-mediated protection extends far beyond enteric and respiratory infections to bacterial sepsis, meningitis, urinary tract infections, necrotizing enterocolitis, ear infections, and allergic dermatitis. The immunoregulatory and anti-inflammatory agents provided by human milk may also decrease the risks of developing various diseases long after weaning. These include certain inflammatory disorders such as asthma, dermatitis, rheumatoid arthritis, diabetes, cardiovascular disease, and certain cancers, as well as obesity and other health problems. For example, in a prospective study of 2,043 Dutch children born in 1996–1997, breast-feeding for more than 4 months was associated with a 33% lower risk of being overweight by age 8 years, as reported by Scholtens et al. in a study published 28 August 2008 ahead of print in *Obesity.* And in a meta-analysis by Owen et al. published in the November 2006 issue of the *American Journal of Clinical Nutrition*, individuals who had been breastfed had a 39% lower risk of developing type 2 diabetes in adolescence or adulthood compared with those who had not.

Goldman notes that some of the nutrients in mother’s milk themselves have strong immunologic properties. “Some of the byproducts of enzymatic digestion of lipids in human milk afford protection against certain bacteria, enveloped viruses, and intestinal parasites such as *Giardia lamblia* and *Entamoeba histolytica*,” he says. “Moreover, human milk provides certain selective bacterial growth factors that support the growth of healthy enteric flora in the infant’s intestines, further enhancing immune competence.” So diverse and integrated are these various components that Goldman regards human milk as containing its own immune system.

The benefits of human milk for human infants are undeniable. But what happens when the nursing infant is exposed to contaminants in human milk? The number of such contaminants is unknown, but the extent of their presence is rapidly growing. Given the potential risks posed by the presence of these toxicants, is there any evidence that the bioactive components of human milk may somehow compensate for these milk-borne pollutants and other toxicants to which a child is exposed?

## POPs at the Tip Top of the Food Chain

Breastfed infants are considered to be at the very top of the food chain for the simple reason that their source of nourishment is other humans, who are already at the top of the food chain. The POPs, which include poly-chlorinated dibenzo-*p*-dioxins (PCDDs), polychlorinated dibenzofurans (PCDFs), polychlorinated biphenyls (PCBs), and certain organochlorine pesticides such as DDT, all tend to become magnified in the food chain over time. Breastfeeding infants are thus the final target of POPs.

**Table:** Other Bioactive Substances in Human Milk

**Growth factors and cytokines** Epidermal growth factor (EGF) Nerve growth factor (NGF) Insulin-like growth factors (IGFs) Tumor necrosis factor-alpha (TNFα) Transforming growth factor-alpha (TGFα) Transforming growth factor-beta (TGFβ) Granulocyte colony–stimulating factor (G-CSF) Interleukins: IL-1β, IL-6, IL-8, IL-10 Prostaglandins Basic fibroblast growth factor (bFGF)
**Hormones** Pituitary hormones (e.g., prolactin, growth hormone, thyroid-stimulating hormone, follicle-stimulating hormone, lutenizing hormone, adrenocorticotropic hormone, oxytocin) Hypothalmic hormones (e.g., thyroid-releasing hormone, somatostatin, prolactin inhibiting and releasing factors) Thyroid and parathyroid hormones (e.g., thyroxine, triiodothyronine, calcitonin, parathormone, parathyroid hormone–related peptide) Steroid hormones (e.g., estradiol, estriol, progesterone, testosterone, 17-ketosteroids, corticosterone, vitamin D) Gastrointestinal peptides (e.g., vasoactive intestinal peptide, gastrin, gastric inhibitory peptide)
**Miscellaneous** Peptides (e.g., sometomedin C) Amino acids (e.g., glutamine) Casomorphins Complement factors

**Adapted from:** Wagner CL, Anderson DM, Pittard WB. 1996. Special properties of human milk. *Clin Pediatr (Phila)* 35(6):283–293.

In 1951 DDT became the first environmental pollutant found in human milk. Since then, DDT and its metabolites have been reported in essentially all human milk tested worldwide. In recent years, additional chemicals have been detected in human milk, among them bisphenol A, polybrominated diphenyl ethers (PBDEs), hexachlorobenzene, and the cyclodiene pesticides, which include dieldrin, heptachlor, and chlordane. Residues of many banned POPs persist in women’s milk.

The persistent lipophilic chemicals found in human milk are preferentially stored in the mother’s adipose tissue. To create milk for her infant, a woman’s body mobilizes lifetime fat stores and therefore transmits a portion of her stores of environmental contaminants to her newborn during breastfeeding. A review by certified nurse–midwife Joanne Jorissen in the October 2007 *Advances in Neonatal Care* notes that on average, the nursling receives about 50 times (per kilogram of body weight) the daily PCB intake of adults, and breastfed infants are predicted to have cumulative PCB exposures that are up to 18% higher than those of formula-fed infants, depending on the duration of breastfeeding.

“During the latter half of gestation there is a redistribution of these chemicals from maternal tissue stores to the milk compartment and to the fetus, as lipids are mobilized for milk production and fetal growth,” says Richard Wang, a medical officer at the National Center for Environmental Health of the Centers for Disease Control and Prevention (CDC). Thus, a woman with a higher body mass index (BMI), which reflects adiposity, will tend to accumulate more chemicals in her body than her leaner counterparts, even if she has the same serum concentration of that chemical or received the same chemical dosage.

“Some of the other factors that can affect the serum concentrations of these chemicals and that need to be considered when interpreting these data among persons include a rapid change in body weight, such as during or after pregnancy, a difference in metabolic clearance, and age,” says Wang. “The latter is an important consideration when dealing with environmental chemicals with lower current emission concentrations than in the past because this difference is likely to contribute to increased amounts of chemicals in persons at increased age.” An older breastfeeding mothers with a high BMI, for example, would tend to pass on larger amounts of chemicals to her infant than would a younger mother with a normal BMI.

Yet, the literature to date supports the idea that the benefits of breastfeeding generally outweigh the hazards posed by infant exposure to POPs in human milk. Most of the data derive from six human cohort studies that have examined the effects of PCBs in human breast milk. Whereas exposures *in utero* may have significant adverse effects on infant development, these studies have suggested that breastfeeding exposures do not. However, several of these studies have indicated that PCBs in human milk can attenuate the developmental benefits of breastfeeding, although not in a statistically significant fashion after controlling for other factors in child development such as parental influence and home environment.

“The fact that studies of child [health] outcomes in highly polluted areas are still better for the breastfed infant . . . would seem to indicate that certain factors in the production of human milk and in the milk itself, immunological and other, may mediate the potential harm of the ambient pollution,” says physician–epidemiologist Miriam Labbok, who directs the Carolina Breastfeeding Institute at the School of Public Health of the University of North Carolina, Chapel Hill. “It would appear that all the experts remain in agreement that there is no reason for WHO to change its breastfeeding recommendations.”

According to Philip Landrigan, director of the Center for Children’s Health and the Environment at Mount Sinai School of Medicine in New York, documented adverse effects on breastfeeding infants—such as impairment of psychomotor development and other neurodevelopmental outcomes—have been seen primarily in cases of high-dose poisonings in which the mother became clinically ill. He says very few data exist on long-term effects of such exposures or on synergistic interactions among chemicals in human milk. “The prospective epidemiologic studies that are needed to assess chronic outcomes that may occur at lower levels of exposure have been undertaken for PCBs but few other persistent chemical pollutants,” says Landrigan.

In her October 2007 review, Jorissen offered this conclusion: “At this point, there is no evidence of a threshold among the general population beyond which the risks of breastfeeding outweigh the benefits, nor is there any evidence demonstrating a clinically significant negative effect of postnatal exposure to PCBs via breast milk. To date, the majority of studies conclude that despite substantially higher PCB loads among breastfed infants, breastfeeding is still preferable to formula feeding.”

Wang points out that many of the environmental chemicals commonly measured in human milk come from the mother’s diet. For example, he says, up to 90% of human exposure to the persistent and lipid-soluble dioxin-like chemicals, including certain PCBs, PCDDs, and PCDFs, is attributed to dietary intake. These chemicals are found at higher concentrations in fatty foods such as red meat, dairy products, and fish. Some of the highest levels of contaminants are seen among women in remote northern areas, such as the Canadian Inuit, who eat a diet rich in seal, whale, and other fatty marine species high on the food chain. Meat eaters in general tend to harbor more POPs than people eating predominantly vegetarian diets.

During gestation and lactation, a woman therefore may change her diet to reduce her infant’s exposure to such chemicals during critical windows of the child’s growth and development. Nursing mothers can also reduce the level of POPs in their milk by maintaining their weight to avoid mobilizing fat stores, says Jenny Pronczuk, a WHO medical officer working in the area of children’s health and the environment—who adds that reducing emissions of POPs into the environment is the long-term solution to this problem and one which risk managers should give greater priority.

## Metals in Mother’s Milk

Lead, mercury, arsenic, cadmium, and other potentially toxic metals that are dispersed throughout the environment also have bioaccumulative features and thus are of concern to the nursing infant. The presence of lead and mercury in human milk has been extensively studied. Both are equally dispersed in the human food chain, and their impact on the nursling’s early development is heavily determined by the mother’s diet and nutritional status. For example, because lead is stored in the bones, breast-feeding mothers who maintain a good calcium intake and healthy bone metabolism during pregnancy are less likely to transfer lead to the infant, according to a review by University of Brasília nutrition professors José G. Dórea and Carmen M. Donangelo in the June 2006 issue of *Clinical Nutrition*.

The mother’s exposure to lead and mercury is more critical during fetal development than during breastfeeding, as the fetus is more vulnerable through placental transfer than through milk. Nonetheless, breast-feeding-mediated exposures to lead and mercury are extremely common. “Lead and mercury reach the nursing infant through very different maternal pathways, and exposures can occur through either human milk or formula milk,” says Dórea. “These days, the infant’s lead burden comes primarily through mother’s milk and infant formula.”

In some instances, Dórea says, commercial formula may be a more serious source of heavy metals than human milk. “Because breastfeeding is essential to a normal, healthy infant development, avoiding breastfeeding and using cow’s milk–based formulas is not a reasonable way to respond to the problem of environmental pollution and human milk contamination.” He adds that the risk of excessive lead exposure for infants, whether breast- or formula-fed, is higher and the effects longer lasting, compared with mercury exposure.

Although numerous studies have found a positive association between breast-feeding and improved cognition, some studies have suggested that exclusive breastfeeding beyond 8 or 9 months might result in lower cognitive scores; harmful substances in human milk and nutritional limitations posed by lack of supplemental feeding (e.g., “table food”) after 6 months are two possible explanations for this observation. In one of the most recent of these studies, conducted at the University of Michigan Center for Human Growth and Development, infants breastfed less than 2 months showed poor neurodevelopmental scores, but infants breastfed exclusively beyond 8 months also showed a decline. “If environmental contaminants are found in human milk, children with long breastfeeding as the sole milk source might have higher levels of toxic substances and be at greater risk for associated developmental ill effects,” authors Clark et al. state in the March 2006 issue of *Ambulatory Pediatrics*.

Nevertheless, Dórea asserts that the neurodevelopmental benefits of human milk tend to override the potential adverse effects of neurotoxicants. “There is much evidence that breastfeeding plays a role in attenuating and reversing exposure to neurotoxic substrates, including lead and mercury,” he says. Breastfeeding may also indirectly affect the metabolism of mercury in exposed infants by increasing elimination of the toxic metal.” Human milk contains many brain-protective substances, including selenium, glutathione, vitamin E, cysteine, tryptophan, choline, taurine, S100B protein, sialic acid, and polyunsaturated fatty acids. Dórea asserts that the nursling’s brain may be protected through the combination of these neuroprotective substances.

One component of human milk that could account for its ability to potentially buffer the nursling from the harmful effects of environmental toxicants is whey protein. Human milk is 80% whey protein, a compound that may greatly increase the body’s endogenous production of glutathione, a ubiquitous cellular antioxidant with many important roles in detoxification and immunity. This helps explain the common experimental finding that tumor prevention by dietary whey protein is accompanied by increased glutathione levels in serum and tissues as well as enhanced immunologic activity.

In addition, the α-lactalbumin in human milk (the bulk of the whey component) has been shown to selectively induce apoptosis in cancer cells. Researchers at Sweden’s Lund University speculate that this mechanism may help purge tumor cells from the gut of the neonate, thereby lowering the incidence of cancer in breastfed individuals, as reported by Svensson et al. in the 11 April 2000 *Proceedings of the National Academy of Sciences*.

## Emergency Feeding of Infants

Malnutrition among infants and young children is presently one of the most severe global public health problems and also among the main reasons the WHO emphatically supports breastfeeding. But when the mother herself is severely malnourished, the nutrient content of her milk may be compromised. “Under many trying conditions, lactation can be robust,” says Goldman. “But there are some limitations when nutrients are limiting, and some of this depends on the type of malnutrition. In mild-to-moderate degrees of protein–calorie deficiency, lactation performance and human milk composition remain satisfactory. In more severe degrees, lactation performance and human milk composition are no longer spared.” He adds that the continuing need for extra calories, protein, and micronutrients due to lactation places an extra burden upon the malnourished woman and may further deplete her body nutrient stores.

Although maternal diet and nutritional status have little influence on the macronutrient (protein, fat, carbohydrate) content of human milk, the situation is different where micronutrients are concerned. “The presence of vitamins and minerals in human milk is directly influenced by a mother’s own nutritional status,” says James Akre, a member of the board of the International Board of Lactation Consultant Examiners, which sets certification standards for the lactation consultant profession. “Micronutrient deficiencies that are believed to be widespread among the world’s women merit continued close attention for the improvement of their own health and that of their infants.”

Goldman summarizes these and other potential risks associated with human milk in volume 54, issue 1 (2007) of *Advances in Pediatrics*. Among them are a lack of certain micronutrients (zinc, iron, and vitamins K, D, and B_12_) in human milk, usually due to inadequacies in the mother’s diet or lack of sun exposure in the case of vitamin D; the presence of foreign food antigens, proinflammatory fatty acids, autoantibodies, and infectious agents such as HIV; and T cells that may colonize immunedeficient infants and thus, for example, may trigger graft-versus-host disease.

Certain deficiencies in micronutrients, notably vitamin B_12_ and vitamin D, may harm the rapidly growing infant before the effect is seen in the adult lactating woman. In the case of vitamin B_12_, neurological damage may result. With vitamin D, the risk of rickets (deformed bones) has recently increased in many parts of the world due to lack of sunlight exposure for the breastfeeding mother and infant [for more information on this link, see “Benefits of Sunlight: A Bright Spot for Human Health,” *EHP* 116:A160–A167 (2008)].

According to Akre, mild-to-moderate subclinical forms of malnutrition are generally not an indication for mothers not to breastfeed their infants. “Not breast-feeding under such circumstances may only worsen the situation for the infant in question, who is deprived of the many benefits of human milk, as well as for the other family members when scarce resources are used to provide a nutritionally adequate substitute,” he says. “We have to keep in mind that adequate nutrition is more critical in early infancy than at any other time in life because of the infant’s high nutritional requirements in relation to body weight and the influence of proper or faulty nutrition during the first months on future health and development. Moreover, the infant is more sensitive to abnormal nutritional situations and less adaptable than in later life to different types, forms, proportions, and quantities of food.” From a nutritional standpoint, he adds, it is far easier to meet the nutritional needs of a mother than those of her nonbreastfed infant.

Many public health officials in the past have recommended the use of commercial formula in emergency situations such as wars or natural disasters. Even here, however, the evidence seems to favor continued breastfeeding as long as it is possible. Labbok cites a study by Jakobsen et al. in the November 2003 issue of *Tropical Medicine & International Health* that used data collected during a 3-month period prior to and during the war in Guinea-Bissau to assess the impact of breastfeeding status on mortality in an emergency. Before the war, there was no significant difference in mortality rates between breastfed and formula-fed infants. During the war, however, the picture changed radically—children who were not breastfed suffered 5–6 times the mortality compared with those who were breastfed.

In countries where infectious diseases account for a large portion of infant mortality, widespread use of commercial formula has resulted in epidemics of diarrhea and respiratory disease. In a study of 9,424 infants and their mothers in Ghana, India, and Peru, researchers found that the risk of dying was 10 times greater in nonbreastfed infants than in predominantly breastfed infants, and double that of partially breastfed infants, as reported by Bahl et al. in the June 2005 *Bulletin of the World Health Organization*. More recently, a major epidemic of diarrheal disease broke out among children under age 5 years when free formula distributed in Botswana—an intervention meant to prevent HIV transmission through mothers’ milk—was mixed with contaminated water, increasing a child’s risk of death by 50 times.

In the September 1991 issue of *Dialogue on Diarrhoea*, nutrition specialist Ted Greiner noted that reconstitution of commercial formula using contaminated water, incorrect water-to-formula proportions, or nonsterilized bottles can lead to diarrhea and other infections in the infant. Milk-based powdered formula can also be contaminated with *Enterobacter sakazakii* and *Salmonella*, prompting the CDC to recommend in 2002 that alternatives to powdered formula be used whenever possible in neonatal intensive care units. The U.S. Food and Drug Administration, moreover, recommends that powdered formula be reconstituted with water at temperatures of at least 158°F to reduce the presence of *E. sakazakii*. (However, in its 5 December 2007 report *EWG’s Guide to Infant Formula and Baby Bottles*, the Environmental Working Group recommends choosing powdered formula over liquid because the packaging for the latter tends to leach more bisphenol A, a chemical the National Toxicology Program concludes may cause adverse brain, behavioral, or prostate gland effects in fetuses, infants, and children.)

According to the Infant Feeding in Emergencies Core Group of the interagency Emergency Nutrition Network, commercial formula should only be used in special circumstances during emergencies, such as when the mother has died or is very ill, or if the mother rejects her infant due to rape or other trauma (temporary formula use may be all that is necessary). “Every effort must be made to re-establish lactation for mothers and babies in such situations,” says Labbok, “and babies born after the start of an emergency should be exclusively breastfed from birth.” The use of commercial formula as a substitute for or complement to human milk tends to divert mothers from the practice of exclusive breast-feeding and undermine their ability to maintain a milk supply, because the amount of milk produced by the mother’s body changes in response to suckling by the infant.

## A Net Gain

After having considered the problem of environmental contaminants in human milk, the WHO, the U.S. Surgeon General, and the American Academy of Pediatrics continue to recommend breastfeeding. “After three decades of study, there is now fairly good evidence that little if any morbidity is occurring from the more common and well-studied chemical agents found in human milk,” says Walter Rogan, a clinical investigator in the NIEHS Epidemiology Branch. “There are very few instances in which morbidity has been described in a nursling that was due to a chemical pollutant in milk.”

Labbok agrees. “To date, no environmental contaminant, except in situations of acute poisoning, has been found to cause more harm to infants than does lack of breast-feeding,” she says. “I have seen no data that would argue against breastfeeding, even in the presence of today’s levels of environmental toxicants.”

Still, Rogan cautions, human milk contains no proven antidote to contaminant exposure. “To the degree that the overall benefits from breastfeeding overlap with the deleterious effects of the chemicals, those benefits might appear to cancel out the harm, but this is hard to study epidemiologically,” he says.

Because of human milk’s nutritional, immunologic, anticancer, and detoxifying effects, Wang, Rogan, and other environmental scientists encourage women to continue the practice of breastfeeding even in the context of widespread pollution. “At the same time,” says Pronczuk, “breastfeeding mothers should be helped and advised on how to avoid alcohol and drugs and remove themselves from polluted environments, while also creating healthier, safer, and cleaner environments for themselves and their children.”

## For More Information

Agency for Toxic Substances and Disease Registry. 2004. Interaction Profile for Persistent Chemicals Found in Breast Milk. Atlanta, GA:Centers for Disease Control and Prevention. **http://www.atsdr.cdc.gov/interactionprofiles/ip03.html**

Committee on Toxicity. 2004. COT Statement on a Toxicological Evaluation of Chemical Analyses Carried Out as Part of a Pilot Study for a Breast Milk Archive. London, United Kingdom:Food Standards Agency. **http://cot.food.gov.uk/cotstatements/cotstatementsyrs/cotstatements2004/cotstatebreastmilk**

Condon M. 2005. Breast Is Best, but It Could Be Better: What Is in Breast Milk That Should Not Be? Pediatr Nurs 31(4):333–338. **http://www.medscape.com/viewarticle/512642_1**

Early Head Start National Resource Center. 2008. Breastfeeding: Guide on On-Line Resources. Washington, DC:U.S. Department of Health and Human Services. **http://www.ehsnrc.org/Publications/Breastfeeding.html**

Environmental Health Perspectives. 2002. Mini-Monograph: Chemical Contaminants in Breast Milk: Impact on Children’s Health. Environ Health Perspect 110:A313–351. **http://www.ehponline.org/**

MedlinePlus. 2008. Breast Feeding. Bethesda, MD:U.S. National Library of Medicine. **http://www.nlm.nih.gov/medlineplus/breastfeeding.html**

Natural Resources Defense Council. 2005. Healthy Milk, Healthy Baby. Chemical Pollution and Mother’s Milk. New York, NY:National Resources Defense Council. **http://www.nrdc.org/breastmilk/chems.asp**

Nickerson K. 2006. Environmental Contaminants in Breast Milk. J Midwifery Womens Health 51(1):26–34. **http://www.medscape.com/viewarticle/522025**

Steingraber S. 2007. The Benefits of Breast Milk Outweigh Any Risks. Los Angeles, CA:Healthy Child Healthy World. **http://healthychild.org/resources/article/the_benefits_of_breast_milk_outweigh_any_risks1/**

## Figures and Tables

**Figure:**
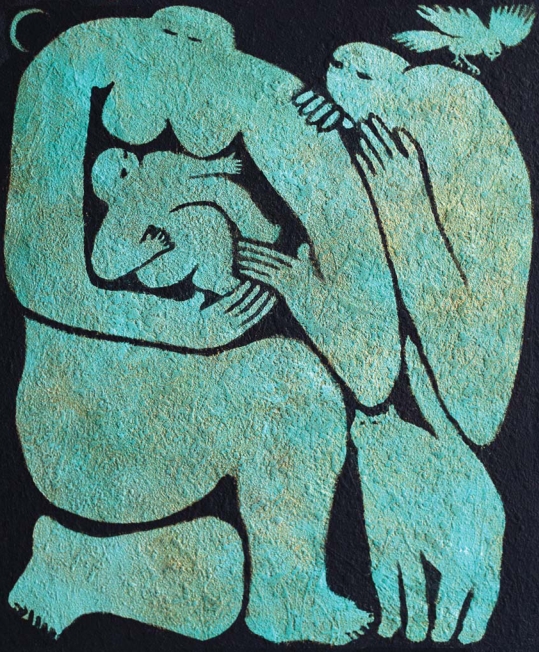


**Figure:**
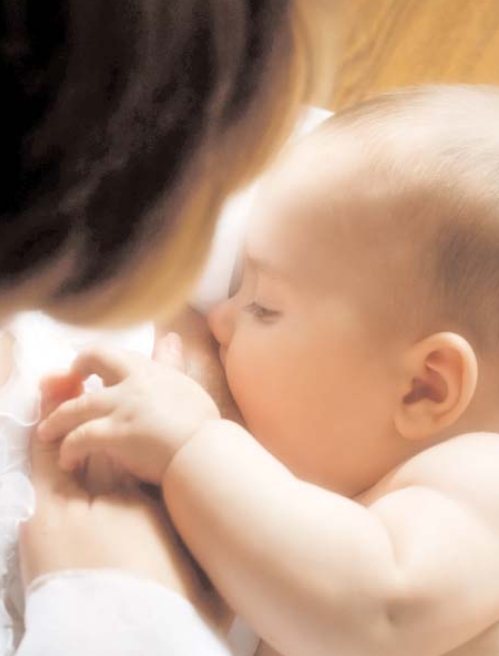
The mother supports the host defense of the infant in two ways. One is via antibodies from her blood that are actively transported over the placenta to the infant’s circulation during fetal life and are ready for use from birth on. The other is due to the numerous and complex defense factors provided via the mother’s milk, available directly after delivery. –Lars Hanson Göteborg University

**Figure:**
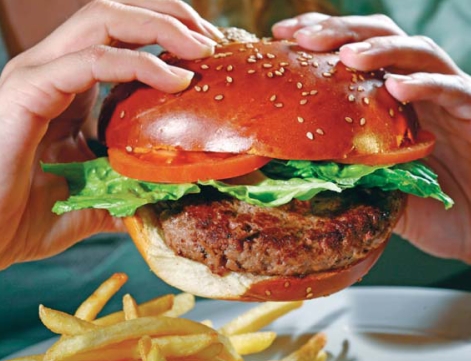
Many of the environmental chemicals that are commonly measured in human milk derive from the mother’s diet. –Richard Wang National Center for Environmental Health Centers for Disease Control and Prevention

**Figure:**
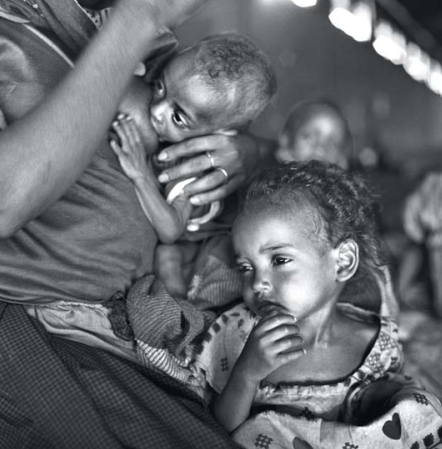
Not breastfeeding [during mild-to-moderate maternal malnutrition] may only worsen the situation for the infant in question, who is deprived of the many benefits of human milk, as well as for the other family members when scarce resources are used to provide a nutritionally adequate substitute. –James Akre International Board of Lactation Consultant Examiners

**Figure:**
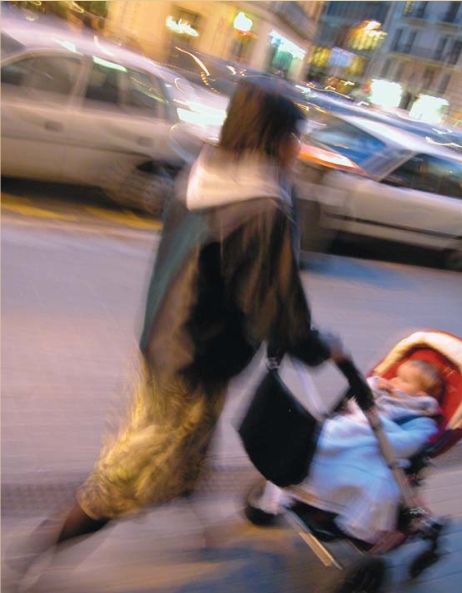
The fact that studies of child [health] outcomes in highly polluted areas are still better for the breastfed infant . . . would seem to indicate that certain factors in the production of human milk and in the milk itself, immunological and other, may mitigate or lessen the potential harm of the ambient pollution. –Miriam Labbok Carolina Breastfeeding Institute University of North Carolina, Chapel Hill

**Figure:**
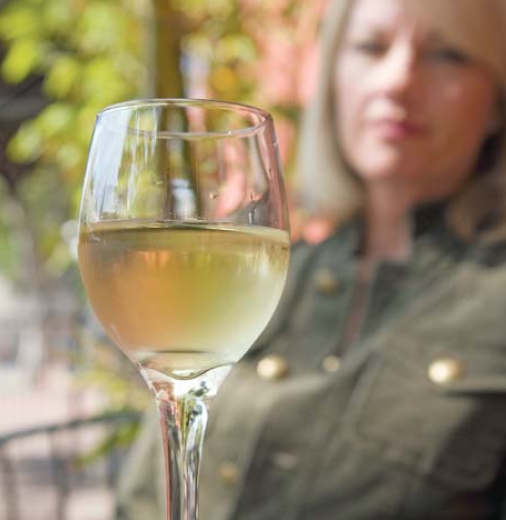
Breastfeeding mothers should be helped and advised on how to avoid alcohol and drugs and remove themselves from polluted environments, while also creating healthier, safer, and cleaner environments for themselves and their children. –Jenny Pronczuk World Health Organization

